# Endothelial nitric oxide synthase: a potential therapeutic target for cerebrovascular diseases

**DOI:** 10.1186/s13041-016-0211-9

**Published:** 2016-03-22

**Authors:** Jinqiang Zhu, Wanshan Song, Lin Li, Xiang Fan

**Affiliations:** State Key Laboratory of Modern Chinese Medicine, Tianjin University of Traditional Chinese Medicine, Tianjin, 300193 P. R. China; Institute of Traditional Chinese Medicine, Tianjin University of Traditional Chinese Medicine, 312 Anshanxi Road, Nankai District, Tianjin 300193 P. R. China; Second Affiliated Hospital of Tianjin University of Traditional Chinese Medicine, Tianjin, 300150 P. R. China

**Keywords:** Nitric oxide, Endothelial nitric oxide synthase, Cerebral blood flow, Cerebrovascular diseases

## Abstract

Endothelial nitric oxide (NO) is a significant signaling molecule that regulates cerebral blood flow (CBF), playing a pivotal role in the prevention and treatment of cerebrovascular diseases. However, achieving the expected therapeutic efficacy is difficult using direct administration of NO donors. Therefore, endothelial nitric oxide synthase (eNOS) becomes a potential therapeutic target for cerebrovascular diseases. This review summarizes the current evidence supporting the importance of CBF to cerebrovascular function, and the roles of NO and eNOS in CBF regulation.

## Background

Cerebrovascular diseases are various vascular diseases of the cerebral circulation. Disrupted arterial oxygen supply to the brain results in cerebrovascular diseases such as transient ischemic attack, stroke, subarachnoid hemorrhage and vascular dementia. Many mechanisms contribute to the complex pathophysiological process. Cerebrovascular diseases have become the major causes of long-term disability and mortality throughout the world.

Currently, the treatment of cerebrovascular diseases includes thrombolytic therapy, anti-platelet aggregation drugs, anticoagulants and neuroprotective agents. However, these treatments all have major limitations, including a short treatment time window, side effects, complications, long-term medication and heavy expenses. Therefore, cerebrovascular diseases are not only substantial medical burdens, but also heavy economic and social burdens. These burdens have stimulated tireless efforts to prevent and cure cerebrovascular diseases, and in particular to define the role of cerebrovascular endothelium in the pathogenesis of cerebrovascular diseases. Cerebrovascular endothelium, as a vascular barrier contacting blood directly, is injured first by vascular risk factors and subsequently forms various cerebrovascular diseases. Interestingly, brain microvascular endothelial cells (BMECs), known as the body’s largest endocrine, paracrine and metabolic organ, can generate and release many vasoactive substances. These include nitric oxide (NO) and endothelin-1 (ET-1), which maintain cerebrovascular homeostasis [1]. In addition, cerebrovascular endothelium plays a critical role in the regulation of cerebral blood flow (CBF). It does so partially through synthesis of NO, a major vasodilator, from L-arginine, by the catalytic reaction of enzyme endothelial nitric oxide synthase (eNOS). Adequate CBF is one of the basic conditions for normal brain function. Endothelial NO production mainly depends on the eNOS activity; therefore, eNOS activation in cerebrovascular endothelium should not be ignored. A recent study has found that in patients exposed to vascular risk factors, endothelium-dependent relaxation dysfunction was detectable before any morphological changes on the vessel wall, implying vascular endothelium is the first to be damaged. More importantly, different risk factors for cerebrovascular diseases including hypertension, hypercholesterolemia, diabetes, aging, smoking, and obesity could all impair contraction and dilation of cerebrovascular endothelium [2]. Therefore, eNOS as an important regulator of cerebrovascular endothelium functions should be recognized as a potential therapeutic target for the prevention and treatment of cerebrovascular diseases.

### Cerebral blood flow and neurological function

The brain consumes 20 % of the body’s energy and nutrients. The basilar artery loop connects to the common carotid artery and is spread over almost all of the brain with numerous branches (the anterior middle and posterior cerebral arteries). The branches extend from soft membrane to the white matter constituting the cerebrovascular system. This system plays an irreplaceable role in supporting the brain by providing it with oxygen and nutrients. Under normal physiological conditions, the brain can maintain adequate CBF through its vascular autoregulatory mechanisms in order to support the energy needs of its cellular constituents. However, many pathological conditions including cerebral ischemia, trauma, stroke, and vasospasm after subarachnoid hemorrhage (SAH) cause abnormal CBF, subsequently inducing diverse brain disorders. Abnormalities in cerebral hemodynamics, such as changes in local perfusion pressure and vascular integrity, are important pathophysiological elements in ischemic stroke. This may further damage neurons or glial cells beyond the initial ischemia injury caused by either hypo- or hyperperfusion [3]. Existing evidence suggests that the cerebrovascular endothelial dysfunction, induced by cerebral ischemia and anoxia-caused vasospasm and/or thrombosis, will further reduce CBF. Continuously decreased CBF can gradually cause learning and memory disorders and cognitive dysfunction, leading to the development of dementia [4–6]. Thus, the regulation of CBF is closely related to neurological function. Dilation of blood vessels is an important way to improve CBF. Vascular endothelial cells regulate the vasodilatation by synthesis and secretion of cytokines, such as PGI_2_ and NO. Studies have shown that the endothelial NO generated in cerebrovascular endothelium is one of the most important signaling molecules of CBF autoregulatory mechanisms [7, 8].

### Endothelial oxide synthase in cerebrovascular regulatory mechanisms

**Fig. 1:**
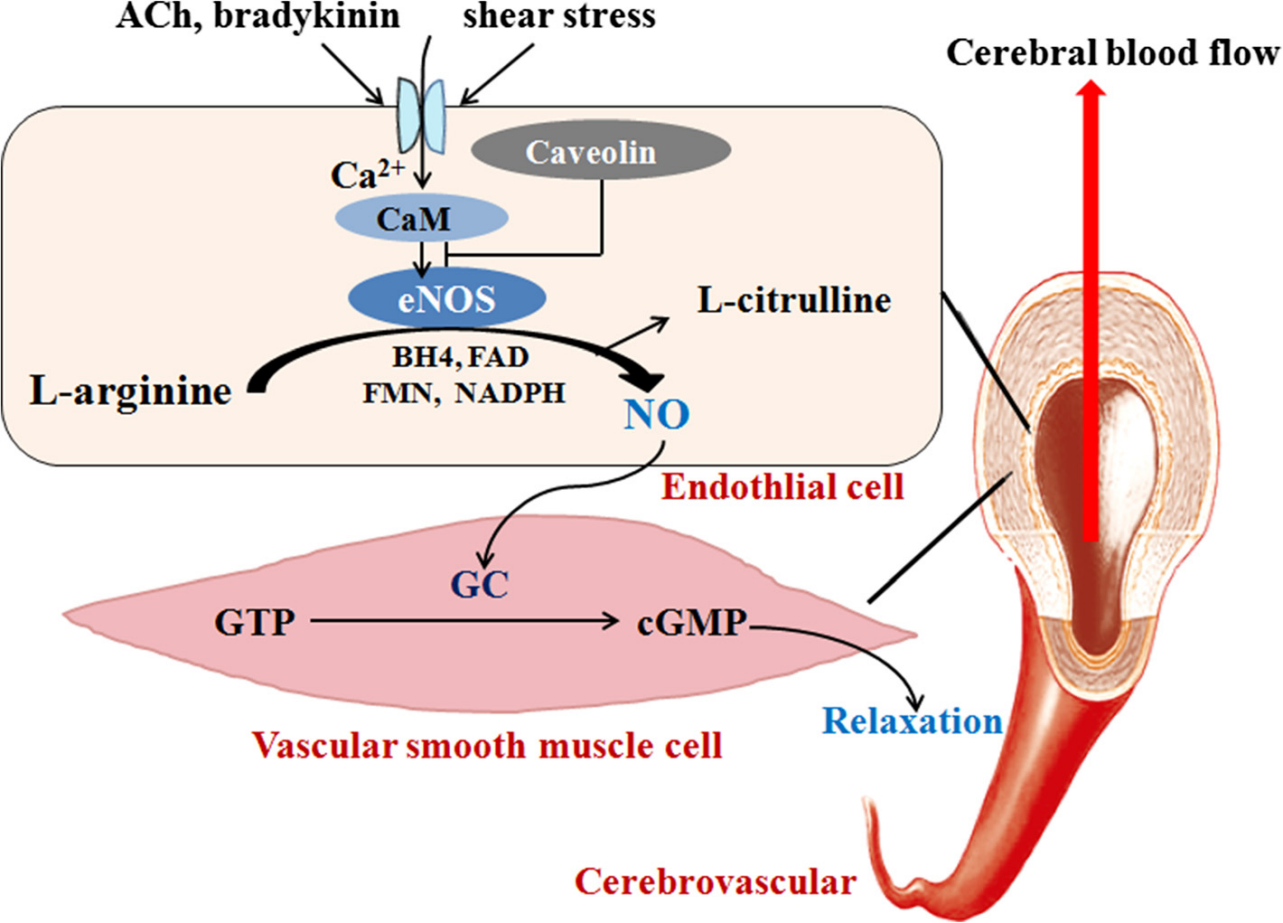
Endothelial oxide synthase (eNOS) and its role in cerebral blood flow (CBF) regulatory mechanisms. eNOS is activated by Acetylcholine (ACh), bradykinin, shear stress, etc., and then catalyzes L-arginine to generate NO. It translates into vascular smooth muscle cells, reacts with guanylate cyclase (GC), and promotes the conversion of guanosine triphosphate (GTP) into cyclic guanosine monophosphate (cGMP), resulting in vascular smooth muscle relaxation and the CBF increase

As mentioned above, the brain is endowed with vasoregulatory mechanisms that assure it receives enough blood to support its energy expenditure. Endothelial NO is one of the most pivotal signaling molecules in these mechanisms (Fig. 1) [7, 8]. It is involved in preserving and maintaining the brain’s microcirculation, inhibiting platelet aggregation, leukocyte adhesion and migration, and reducing smooth muscle proliferation. NO plays a pivotal role in regulating cerebrovascular effects by increasing or decreasing oxygen, and elevating carbon dioxide, carbon monoxide and cerebrovascular autoregulation [9, 10]. It is generally acknowledged that the loss of endothelial NO is the central mechanism in the pathogenesis of endothelial dysfunction. In both cerebral and peripheral vasculatures, reduced availability of endothelial NO will result in major detrimental alterations of vascular functions. These include vasoconstriction, an increase in arterial blood pressure, proliferation of vascular smooth muscle cells (VSMCs), platelet aggregation, white blood cell adhesion, and inflammation. Reduced availability of endothelial NO plays an essential role in the initiation and progression of vascular diseases such as atherosclerosis. Therefore, preserving endothelial NO production is an important strategy for the prevention of cerebrovascular diseases [11]. However, diverse vascular risk factors can directly or indirectly reduce endothelial NO production. Oxidative stress caused by excessive production of reactive oxygen species (ROS) and primarily superoxide anions is considered to be the most important mechanism of reducing endothelial NO. On one hand, vascular risk factors promote the up-regulation of NADPH-oxidase activity, generation of superoxide anions, chemical inactivation of NO, and generation of a potent oxidant, peroxynitrite [12]. On the other hand, oxidative stress induced by increased peroxynitrite production may oxidize tetrahydrobiopterin (BH4), an essential co-factor required for eNOS activity. If the concentration of BH4 becomes suboptimal, the eNOS dimer will uncouple from other eNOS monomers, resulting in reducing the production of endothelial NO and increasing the production of superoxide anions and peroxynitrite [13]. It is reported that decreased arterial oxygen supply (hypoxia) increases the CBF baseline in humans [14]. Thus, NO plays a significant role in both the signaling event and the CBF response [15]. Conversely, nonselective NOS inhibition abolishes the CBF increase response to hypoxia in rats [16]. It follows that the NO signaling pathway plays a central role in the CBF response to these alterations, and this post-ischemia pathway dysfunction causes dysregulation of these normal physiological responses. Recent studies on CBF regulation by endothelial NO have provided new insights about the physiological control of cerebrovascular functions and the pathological mechanisms of neurological diseases. They also supply new clues for developing novel therapeutic strategies for cerebral dysfunction [9]. Indeed, eNOS has been paid even more attention than NO because of the instability of NO and the regulatory mechanisms of eNOS on NO production.

### Endothelial nitric oxide synthase and cerebrovascular diseases

#### eNOS function

Innate eNOS activity determines susceptibility to injury due to its conferring protection against secondary neuronal damage. Impairment of eNOS activity by SAH, traumatic brain injury (TBI) and ischemic stroke has been implicated in many cellular mechanisms of neuronal injury [17].

Delayed cerebral ischemia (DCI) is one of the major causes of morbidity and mortality following SAH. A recent study has demonstrated that the pathogenesis of DCI involves the NO signaling pathway [18]. Meanwhile, genetic variation in the eNOS gene in humans influences the risk of DCI after SAH, because T-786C single nucleotide polymorphism of the eNOS gene promoter can cause lower eNOS activity and result in greater risk [19, 20].

eNOS also plays an important role in maintaining CBF after TBI. Patients with decreased endogenous eNOS levels have poorer outcomes than patients with alleles that do not affect eNOS levels [21]. A previous study has suggested that the immunoreactivity of eNOS is increased in the microvasculature surrounding the area of impact in the first 3 days after experimental trauma [22]. However, eNOS knockout mice have greater reduction in CBF than wildtype variants in the first 2 h after trauma [23]. Genetic variants of eNOS also influence the maintenance of CBF after severe TBI.

Ischemic stroke is most often caused by a thrombotic or embolic blockage of a cerebral artery, which causes blood flow interruption and tissue death. Studies have found that thrombotic cerebral infarctions appear in eNOS+/− mice as early as 3–6 months of age. eNOS knockout mice have bigger infarcts than wildtype variants after ischemic stroke caused by middle cerebral artery occlusion (MCAO) [24, 25]. In contrast, the administration of NO precursor (L-arginine) or donors (sodium nitroprusside (SNP) and 3-morpholino sydnonimine) to rat MCAO models of focal ischemia have improved CBF, prevented cerebral tissue necrosis and decreased brain ATP and glutamate levels. This appears to be time-dependent and limited to the first 30 min after ischemic onset. During this period, NO elicits vasodilatory effects, improves CBF and reduces the infarct size, while it evokes neurovascular toxicity beyond this point [25–27]. Moreover, eNOS not only promotes vasodilation but also increases the proliferation and migration of VSMCs, thereby enhancing arteriogenesis after stroke [28]. Activation of eNOS mediates protection from stroke by preserving CBF and inhibiting inflammation, platelet aggregation, thrombosis, and apoptosis [11]. Continuous voluntary running confers long-term upregulation of eNOS in the vasculature and higher numbers of circulating endothelial progenitor cells (EPCs) in the blood. This nevertheless enhances neovascularization and CBF by eNOS-dependent mechanisms after brain ischemia [29]. eNOS is also essential for mobilization of EPCs. The impaired neovascularization in mice lacking eNOS is related to a defect in progenitor cell mobilization [30].

Uncoupled eNOS in the cerebral arteries has been reported to participate in hypoxic-ischemic brain injury by inducing oxidative stress under ischemia [31]. Uncoupling of eNOS emerges from an absence of either its substrate L-arginine, and/or co-factor BH4, which is required for coupling of the two functional eNOS subunits. This is known to be a common phenomenon in many diseases, such as hypertension, diabetes, and hypercholesterolemia [32, 33]. In an uncoupled state, the electron flow inside the eNOS peptide is diverted to the molecular oxygen, thereby leading to an uncontrolled eNOS uncoupling and enhanced O_2_^•-^ production. In summary, normal eNOS function is vital to alleviate cerebrovascular diseases.

#### Regulatory mechanisms of eNOS activity

eNOS has a homologous dimerized structure consisting of two identical subunits. Each subunit contains a C-terminal reductase domain and an N-terminal oxygenase domain. The former contains binding sites for NADPH, flavin adenine dinucleotide (FAD), and flavin mononucleotide (FMN), in close homology with cytochrome P-450 reductase; the latter binds heme, BH4, and the substrate L-arginine. Between these two regions lies the calmodulin (CaM) binding domain, which plays a key role in both the structure and function of the enzyme. To obtain full catalytic activity under physiological conditions, separate N-terminal oxygenase and C-terminal reductase domains of eNOS must be joined together in the presence of heme, BH4 and Ca^2+^/CaM complex [7, 34]. Many studies suggest that the enzyme is generally activated by an increase in intracellular Ca^2+^ via either influx of extracellular Ca^2+^ or release from intracellular storage sites. However, eNOS activation is also independent of Ca^2+^, while activated by mechanical forces, including shear stress, cyclic strain, and G protein [35–37].

**Fig. 2:**
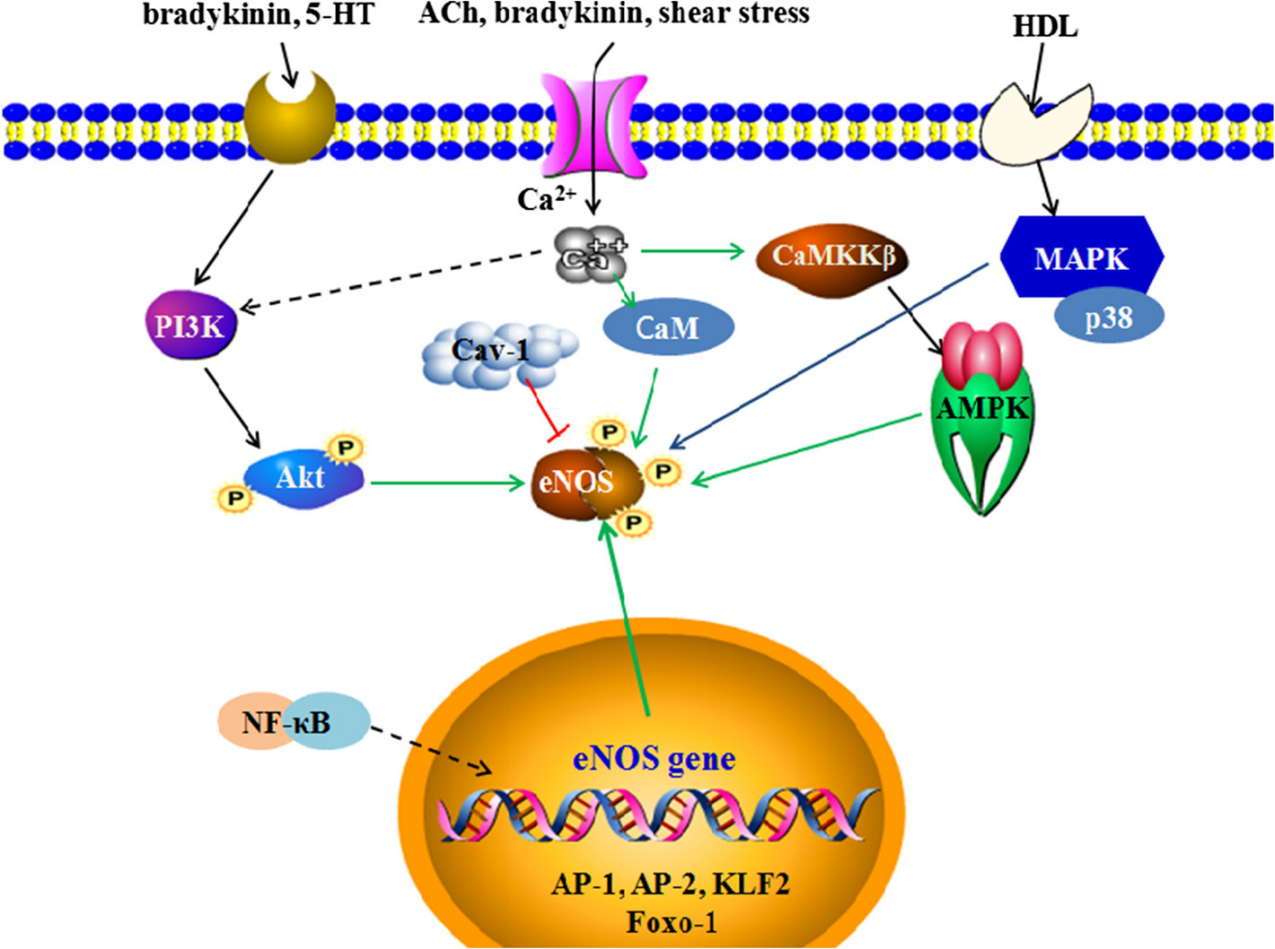
The regulatory mechanisms of eNOS activity. The mechanisms work at both the genetic and protein level. At the genetic level, nuclear factor-κB (NF-κB), activator protein-1 (AP-1), activator protein-2 (AP-2), Krűppel-like Factor 2 (KLF2), endothelin family and Forkhead box O1 (Foxo-1) genes regulate eNOS expression; at the protein level, several factors, such as bradykinin, 5-hydroxytryptamine (5-HT), ACh, shear stress, and high density lipoprotein (HDL), promote eNOS phosphorylation by PI3K/Akt, AMPK and MAPK pathways. In addition, binding with calmodulin (CaM) enhances eNOS activation, while caveolin-1(Cav-1) inhibits eNOS activation

The regulatory mechanisms of eNOS activity are extremely complex, and can be divided into the genetic and protein level (Fig. 2). At the genetic level, the expression and stability of eNOS genes are related to eNOS activity. The eNOS promoter contains a large number of sites for the binding of transcription factors, including activator protein-1 (AP-1), activator protein-2 (AP-2), endothelin family, nuclear factor-κB (NF-κB), and neurofibromin 1 (NF-1). These transcription factor complexes can regulate eNOS expression. In addition, hypoxia, lipopolysaccharide and several cytokines can induce the expression of two kinds of eNOS cytoplasmic proteins. They bind to the 3′ untranslated region of eNOS mRNA rich in cytosine, subsequently leading to configuration changes and RNA enzyme activation, which cause lower eNOS mRNA stability and shorter half-life. Many factors, such as hypoxia, estrogen, and exercise are now known to affect eNOS expression [38–40]. At the protein level, regulatory mechanisms mainly include eNOS translocation, complex formation and the phosphorylation of the amino acid residues. The association of eNOS with bradykinin B2 receptor and caveolin-1(Cav-1) in the caveolae and microdomains of the endothelial plasma membrane leads to inhibition of eNOS activity. This inhibition is apparently the result of functional interference with CaM binding and electron transfer [41, 42].

Cav-1 on cell membrane binds to not only the oxygenase domain but also the reductase domain of eNOS. Binding of Cav-1 to the eNOS reductase domain inhibits the combination of heme iron with eNOS, and prevents electron transfer from the reductase. In eNOS the Cav-1 binding motif lies between the heme and the CaM binding domains adjacent to a glutamate residue (Glu-361), a necessary site for the binding of L-arginine [42–44]. Thus, Cav-1 binding to eNOS inhibits the trafficking and activation of eNOS. Two new-found proteins, eNOS interacting protein (NOSIP) and eNOS traffic inducer (NOSTRIN), have been confirmed to interact with eNOS and Cav-1 to form a ternary complex. The complex then induces eNOS migration from the plasma membrane and significantly reduces eNOS activity [45–47].

Present studies show that eNOS phosphorylation occurs in its serine, threonine and tyrosine residues. Within these residues, the phosphorylation of the Thr495, Ser633 and Ser1177 sites have the most significant influences on eNOS activity. The phosphorylation of the Thr495 site prevents CaM binding to eNOS, thereby reducing eNOS activity [48], while the phosphorylation of the Ser1177 site enhances the catalytic ability of eNOS through inhibiting the separation of CaM and eNOS, and increases the internal electron transfer rate of eNOS; the phosphorylation of the Ser633 site also enhances eNOS activity [49–51]. The threonine protein kinase (Akt/PKB) is an important determinant of the phosphorylation of the eNOS Ser1177 site, which is involved in the basic activation of eNOS and its agonist activation [52, 53].

Akt mainly resides in the cytoplasm inactively; translocation to the cell membrane induces its activation and the phosphorylation of eNOS [54]. It is directly controlled by the phosphatidylinositol-3 kinase serine (PI3K)-dependent phosphorylation pathway. PI3K recruits Akt to cell membrane and phosphorylates it [55]. Bradykinin and 5-hydroxytryptamine (5-HT) act with membrane receptors, leading to the increase of cytoplasmic Ca^2+^ concentration and activation of CaM. It first promotes eNOS uncoupling with Cav-1; eNOS then couples with CaM to form an eNOS/CaM complex and translocates to the cytoplasmic membrane. After translocation, the eNOS/CaM complex induces phosphorylation of eNOS amino acid residues at the Ser1177 site and dephosphorylation at the Thr495 site through the Akt/PKB pathway, resulting in eNOS activation [56–58].

Meanwhile, after cytoplasmic Ca^2+^ concentration increases, Ca^2+^/calmodulin- dependent protein kinase kinase-β (CaMKKβ), an alternative upstream kinase for adenosine 5′-monophosphate (AMP)-activated protein kinase (AMPK) and a key molecule of the AMPK signaling pathway, induces the phosphorylation of AMPK at the Thr172 site. Upon activation, AMPK promotes the phosphorylation of eNOS at the Ser1177 and/or Ser633 sites, enhances eNOS activity and promotes NO synthesis. It is restrained by Compound C, a specific inhibitor of AMPK [59–63]. Interestingly, eNOS Ser1177 phosphorylation has dual functions after ischemic stroke. eNOS expression, eNOS Ser1177 phosphorylation and eNOS monomer formation is increased in the acute phase, but Ser1177 phosphorylation is markedly decreased in the later phases of ischemia. Uncoupling of eNOS is significantly increased in this phase. Because eNOS dysfunction contributes to secondary injury and inhibits tissue repair, dephosphorylation and monomer formation of eNOS may become a potential therapeutic target of cerebral ischemia in the later phases [64]. The Ser1177 site may be the more important targeting mechanism compared with the Ser633 site.

Furthermore, eNOS can be activated by either shear stress through phosphorylation of Ser1177 via the PI3K/Akt pathway [50], or by high density lipoprotein (HDL) modification by Akt kinase and mitogen-activated protein kinase (MAPK) [65].

In summary, the Ser1177and Ser633 sites may be potential therapeutic target sites. Regulating the phosphorylation of these two sites and the eNOS activation are regulatory mechanisms for CBF.

### The candidates for eNOS targeted therapy

Recent studies have demonstrated that statins have a potential to enhance the bioavailability of endothelium-derived NO, principally by up-regulating and activating eNOS. This is mediated by diverse signaling, such as Rho/ROCK, PI3K/Akt, caveolin, ERK 1/2, and so on [66, 67]. Similarly, atorvastatin and simvastatin could increase eNOS expression by inhibiting Rho-kinase and increasing Ras-mediated activation of the PI3K-Akt and ERK1/2-RSK signaling pathways, respectively [68, 69]. Additionally, the Rho-kinase inhibitor fasudil ameliorates brain tissue injury via rapid augmentation of eNOS phosphorylation, suppressing eNOS dephosphorylation and monomerization in the ischemic brain [64].

## Conclusion

NO is an important mediator in the regulation of CBF. However, the NO signaling pathway influences the evolution of multiple aspects of secondary brain injury and interacts with many other signaling pathways, which makes it difficult to translate results from animal models to patients. A systematic review of NO donor administration during animal stroke models demonstrates an overall improvement in CBF and a decrease in infarction volume [70]. However, whether these results can be translated into patients with cerebral ischemia/reperfusion injury (e.g. ischemic stroke, cardiac arrest) has not yet been tested [71]. It is also likely that many of the neuroprotective effects of NO donors are the result of not only the improvement of CBF, but also the NO donors’ metabolic and cellular effects.

Therefore, it is difficult to use direct administration of NO donors to achieve the expected therapeutic efficacy. As a key enzyme of NO synthase, eNOS becomes a potential therapeutic target for the prevention and treatment of cerebrovascular diseases. There is numerous evidence to suggest that NO derived from eNOS is neuroprotective after an acquired brain injury. The modulation of eNOS during and/or following ischemic injury often represents a futile compensatory mechanism due to a significant decrease in NO bioavailability, coupled with a dramatic increase in the ROS levels that further neutralize NO [19]. Thus, the modulation of eNOS used in combination with an antioxidant may be significantly more effective. A better understanding of the roles of eNOS might lead to the development of exciting new pharmacotherapies to mitigate secondary brain injury, with the aim of reducing the morbidity and mortality from cerebrovascular diseases. 

### Ethics approval and consent to participate

Not applicable.

## Abbreviations

5-HT: 5-hydroxytryptamine
Ach: acetylcholine
Akt: protein kinase B
Akt/PKB: threonine protein kinase
AMP: adenosine 5′-monophosphate
AMPK: adenosine 5′-monophosphate (AMP)-activated protein kinase
AP-1: activator protein-1
AP-2: activator protein-2
BH4: tetrahydrobiopterin
BMECs: brain microvascular endothelial cells
CaM: calmodulin
CaMKKβ: Ca^2+^/calmodulin-dependent protein kinase kinase-β
Cav-1: caveolin-1
CBF: cerebral blood flow
cGMP: cyclic guanosine monophosphate
DCI: delayed cerebral ischemia
eNOS: endothelial nitric oxide synthase
ET-1: endothelin-1
FAD: flavin adenine dinucleotide
FMN: flavin mononucleotide
Foxo-1: Forkhead box O 1
GC: guanylate cyclase
GTP: guanosine triphosphate
HDL: high density lipoprotein
KLF2: Krűppel-like Factor 2
MAPK: mitogen-activated protein kinase
MCAO: middle cerebral artery occlusion
NF-1: neurofibromin 1
NF-κB: nuclear factor-κB
NO: nitric oxide
NOSIP: eNOS interacting protein
NOSTRIN: eNOS traffic inducer
PI3K: phosphatidylinositol-3 kinaseserine
ROS: reactive oxygen species
SAH: subarachnoid hemorrhage
SNP: sodium nitroprusside
TBI: traumatic brain injury
VSMCs: vascular smooth muscle cells
